# Uromodulin concentrations are not associated with incident CKD among persons with coronary artery disease

**DOI:** 10.1186/1471-2369-12-2

**Published:** 2011-01-14

**Authors:** Michael G Shlipak, Yongmei Li, Caroline Fox, Josef Coresh, Carl Grunfeld, Mary Whooley

**Affiliations:** 1 Division of General Internal Medicine, San Francisco VA Medical Center; San Francisco, CA, USA; 2 Department of Medicine University of California, San Francisco; San Francisco, CA, USA; 3 National Heart, Lung and Blood Institute's Framingham Heart Study, Framingham, MA; Division of Endocrinology, Metabolism, and Diabetes, Department of Medicine, Brigham and Women's Hospital and Harvard Medical School, Boston, MA (CSF), USA; 4 Department of Epidemiology, Johns Hopkins Bloomberg School of Public Health, Baltimore, MD, USA; 5 Division of Metabolism and Endocrine Sections, San Francisco VA Medical Center; San Francisco, CA, USA

## Abstract

**Background:**

A common variant of the UMOD gene was linked with prevalent chronic kidney disease (CKD) in large, genomics consortia. One community-based study found that urine concentrations of the uromodulin protein forecast risk of incident CKD. This study within persons with known coronary artery disease (CAD) evaluated whether uromodulin concentrations could distinguish CKD risk.

**Methods:**

In the Heart and Soul Study, the UMOD snp (12917707) was genotyped in 879 individuals with baseline creatinine clearance (CrCl) measured from a 24-hour urine collection. Uromodulin protein was measured from stored urine specimens among a subset of 120 participants, balanced by genotype. Incident CKD cases (N = 102) were defined by an initial CrCl > 70 ml/min and a 5-year follow-up CrCl <60 ml/min; controls (N = 94) were matched on age, sex, and race.

**Results:**

Among 527 self-described White participants with DNA, 373 (71%) were homozygous for the dominant allele (G/G), 133 (25%) were heterozygous (G/T) and only 21 (4%) were homozygous for the minor allele (T/T). The T/T genotype had an approximately 11 ml/min higher CrCl than the other 2 groups, but this difference did not reach statistical significance (p = 0.20). The T/T genotype had significantly lower uromodulin levels than the common G/G genotype, and the G/T genotype had intermediate levels. However, uromodulin concentrations were similar between cases and controls (44 vs. 48 mg/dL, p = 0.88).

**Conclusions:**

This study among a cohort of persons with established CAD found no association between urine uromodulin and incident CKD, although UMOD genotype was associated with urine uromodulin concentrations.

## Background

A common variant in the region of the *UMOD *gene was recently discovered in association with chronic kidney disease (CKD) in the Cohorts for Heart and Aging Research in Genomic Epidemiology (CHARGE) Consortium of 19,877 participants of European ancestry.[1] This finding was further validated in the CKDGen collaboration of 67,093 participants, also of European ancestry.[1,2] Each copy of the minor allele (frequency of 0.18) was associated with 20% lower odds of CKD across these population-based studies. Prior studies have found that rare mutations in the *UMOD *gene cause at least two forms of autosomal dominant hereditary kidney disease that progress into end-stage renal disease (ESRD) (familial juvenile hyperuricemicnephropathy and a form of medullary cystic kidney disease). The *UMOD *gene codes the protein uromodulin, also known as Tamm Horsfall Protein (THP), which is the most abundant urinary protein in healthy individuals. In the familial disorders with mutations in the *UMOD *gene, a mutant uromodulin protein is produced that is retained in the endoplasmic reticulum, leading to decreased expression on the apical plasma membrane and decreased levels in the urine.[3,4]

To evaluate urine uromodulin concentrations as an indicator of CKD risk, CHARGE investigators recently measured uromodulin levels in the Framingham Heart Study (FHS) and the Atherosclerosis Risk in Communities (ARIC) Study.[5] In both FHS and ARIC the protective genotype was associated with *lower *urine concentrations of uromodulin. In a matched case/control design for incident CKD in FHS, baseline concentrations of uromodulin were significantly and independently higher among participants who subsequently developed CKD over 10 years of follow-up, as defined by a creatinine-based estimated glomerular filtration rate (eGFR) < 60 ml/min/1.73 m^**2**^. Whether or not the findings from FHS, a community-based study, extend across other settings is an important question to understand the potential of uromodulin protein as a biomarker for kidney disease onset and progression.

Patients with cardiovascular disease are a population at high risk for developing CKD [6,7]. Atherosclerosis in both large arteries and in the microvasculature appears to contribute to declining kidney function [8,9]. In the Heart and Soul Study, a well-characterized cohort of persons with established coronary artery disease (CAD), we evaluated whether baseline urine uromodulin concentrations would distinguish participants with and without subsequent progression to CKD after 5 years of follow-up.[10] In addition, we evaluated whether *UMOD *genotype was associated with urine concentrations of uromodulin in these participants.

## Methods

### Heart and Soul Description

The Heart and Soul Study is an observational study originally designed to investigate the influence of psychosocial factors on the progression of CAD. Methods have been described previously.[10] Briefly, participants were recruited from outpatient clinics in the San Francisco Bay area if they met one of the following inclusion criteria: history of myocardial infarction, angiographic evidence of >50% stenosis in ≥1 coronary vessels, evidence of exercise-induced ischemia by treadmill or nuclear testing, history of coronary revascularization, or documented diagnosis of CAD by an internist or cardiologist. Participants were excluded if they were not able to walk 1 block, had experienced myocardial infarction within the past 6 months, or were likely to move out of the area within 3 years. The study protocol was approved by the Institutional Review Boards of participating institutions, and all participants provided written informed consent. Between September 2000 and December 2002, 1024 participants enrolled and underwent a day-long baseline study appointment that included a medical history, physical examination, and comprehensive health status questionnaire. Of these, 982 participants provided DNA samples, and 879 of these provided 24-hour timed urine collections. After 5 years of follow-up, all surviving participants were invited to return for a repeat examination. Of the original 1024 enrollees, 195 had died before the 5-year examination. Between September 2005 and December 2007, 667 (80%) of the surviving 829 participants completed the 5-year follow-up examination.

### Design

The initial step of this study was to genotype the relevant *UMOD *sNP (rs12917707) identified from the CHARGE analysis across all Heart and Soul participants with available stored DNA and 24-hour urine samples (N = 879). In study 1, we evaluated the cross-sectional association of genotype with baseline creatinine clearance among all persons with available urine samples. In study 2, we evaluated whether the genotype at sNP (rs12917707) was associated with urine concentrations of uromodulin, as previously described in FHS and ARIC. In this analysis, we measured uromodulin concentrations among all participants who were homozygous for the minor allele (T/T, n = 24) and among 48 participants each who were heterozygous (G/T) or homozygous for the dominant allele (G/G). In study 3, we used a nested case-control design to determine whether urine uromodulin levels were associated with incidence of CKD. We identified cases defined by an initial measured creatinine clearance (CrCl) of > 70 ml/min and CrCl < 60 ml/min after 5 years of follow-up. For each case participant, a control was selected who was matched on age, sex, and race. We chose not to match on urine albumin excretion, so that we could compare it with urine uromodulin as a predictor of incident CKD. Urine uromodulin levels were then measured from baseline stored specimens with the technician blinded to case and control status. Stored urine specimens were available from 102 of the cases and 94 controls.

### Genotyping Methods

Of the 1024 participants, 982 individuals provided DNA for analysis. The SNP marker for rs12917707 was genotyped using TaqMan^® ^SNP Genotyping Assays (Applied Biosystems, Foster City, CA, http://www.appliedbiosystems.com) functionally tested by Applied Biosystems and available on demand. TaqMan^® ^PCR reactions were done with Universal Master Mix Amperase^® ^UNG, 0.083 uL Taqman 40× probe mix and 1.417 uL of water, 1 ul of DNA normalized to 10 ng/ul, for a 5 uL total volume. The PCR conditions for the TaqMan^® ^SNP Genotype Assays were: one enzyme activation step at 95.0°C for ten minutes, and 50 alternating cycles of denaturation at 95.0°C for 15 seconds and reannealing and extension at 60.0°C for one minute. All PCR reactions and allelic discrimination reactions were performed on an ABI 7900HT Real-Time PCR System (Applied Biosystems, Foster City, CA) and analyzed using SDS 2.3 software (Applied Biosystems, Foster City, CA).

### Kidney Function Methods

Creatinine clearance (CrCl) was measured by a 24-hour urine collection in all participants. At the intake appointment, participants were provided with a 3-L collection jug and instructed to save all urine between the end of their intake appointment and the time when a researcher recovered the urine. Participants were instructed to keep the urine collections refrigerated at all times. Research personnel arrived at the participants' home 24 h after their inception appointments to ensure accurately timed specimens. At that time, participants were asked about the time of their first and last voids. When more than 1 h had passed since their last void, participants were instructed to void at that time to complete the collection. All participants were asked whether they were able to collect all urine or whether some fraction had been inadvertently discarded. When the sample was reported to be incomplete, participants were asked to repeat the collection, and research personnel returned 24 h later to re-collect the urine. When the 24-h urine volume was <1 L, participants were asked to repeat the collection to ensure an adequately collected specimen. Similarly, when the 3-L collection jug was completely full, participants were given two new jugs and asked to repeat the collection to ensure that no urine was inadvertently discarded. When participants were unable to collect all urine for any reason or had urinary incontinence, their samples were deemed inadequate and no data were recorded for these participants. CrCl was calculated using the following formula: urine creatinine (mg/dl) * 24-h urine volume (dl)/serum creatinine (mg/dl) * 1440 (min/d). This procedure was repeated at the 5-year follow-up visit. Baseline kidney function was also estimated by GFR equations using either creatinine[11] or cystatin C[12].

### Uromodulin Measurement Methods

Uromodulin was measured by the method of Lau et al with slight modifications.[13] 96-well microtiter plates were coated with 100 μl of 10 μg/ml lectin WGA overnight at 4°C. Plates were washed and blocked with 200 μl blocking buffer (3% BSA in PBS) for 2 hours at room temperature, then washed again and allowed to dry at 37°C for 3 hours. After cooling to room temperature, plates were sealed and stored at 4°C. Urine samples and standards (Biomedical Techologies, Stoughton, MA) were diluted in TEA buffer (0.5% triton X-100, 20 mM EDTA, pH 7.5) and added to wells in duplicate. After one hour incubation at 37°C, wells are washed and anti-uromodulin antibody (Biomedical Techologies, Stoughton, MA) was added. After one hour incubation at 37°C, wells were washed and goat anti-rabbit IgG horseradish perxiodase (Bio-Rad, Hercules, CA) was added. After 1 hour incubation at 37°C, wells are washed and color was developed by the addition of TMB substrate solution and incubation at room temperature for 15 minutes. The reaction is stopped by adding 2N H_2_SO_4, _followed by reading immediately at OD_450 _and OD_620_. Urinary uromodulin concentration was determined by referring to standard curve.[13]

### Statistical Analysis

The analysis began with Study 1, the comparison of *UMOD *genotype with baseline characteristics among the 879 participants with available DNA. These comparisons were stratified by race- Whites and non-Whites. Characteristics included demographics (age, sex, race), body mass index, prevalent hypertension and diabetes, and the urine albumin to creatinine ratio (ACR). Unadjusted comparisons were made by the Kruskall-Wallis test, Chi-square, or Fisher's exact test. We next compared baseline kidney function across genotype categories, using measured CrCl and estimated GFR by cystatin C and creatinine, separately. Because the T/T genotype group appeared to differ compared with the G/G and G/T groups, we also compared T/T versus all others using non-parametric statistics.

Our next step, Study 2, was to compare urine uromodulin concentrations by genotype using unadjusted and adjusted linear regression with log-transformed urine uromodulin as the outcome (N = 120). These analyses were repeated with restriction to Whites only. For Study 3, we compared uromodulin levels between incident CKD cases and controls (N = 196). The median uromodulin concentration, uromodulin-to-creatinine ratio (UCR), and total daily uromodulin were compared by descriptive statistics. We categorized both uromodulin and UCR into quartiles defined using cutpoints from the control group; the distribution of cases and controls across quartiles was evaluated using the Chi-square statistic. Then, we evaluated uromodulin, UCR levels, and total uromodulin as continuous variable predictors (log-transformed per SD) of case-control status using multivariate conditional logistic regression, adjusted for demographic characteristics, body mass index, hypertension, and diabetes.

## Results

Among the 527 self-described White participants with DNA, 373 (71%) were homozygous for the dominant allele (G/G), 133 (25%) were heterozygous (G/T) and only 21 (4%) were homozygous for the minor allele (T/T). The small T/T group was somewhat younger than the G/G and G/T genotype groups (Table 1). Baseline kidney function appeared better in the T/T group compared with the large G/G and GT groups, as we previously observed.[1] The T/T genotype had an approximately 11 ml/min higher CrCl, and 8 ml/min/1.73 m^2 ^higher eGFRcys, and 4 ml/min higher eGFRcr; none of these differences reached statistical significance. Baseline kidney function was similar between the G/G and G/T genotypes. Among 352 non-Whites, 88% were the dominant G/G genotype, 11% were heterozygous, and only 1% (N = 3) were homozygous for the minor allele. Characteristics across these categories are shown in Table 1.

**Table 1:** Characteristics of Participants in the Heart and Soul Cohort by Genotype

Characteristics	G/G (n = 683)	G/T (n = 172)	T/T (n = 24)	P value	P Value TT vs. Other
**Whites**					

**N**	373	133	21	-	-
**Age**	68 ± 11	69 ± 10	64 ± 12	0.24	0.20
**Male**	86%	86%	86%	0.99	0.58
**Body Mass Index**	29 ± 5	29 ± 6	30 ± 6	0.48	0.23
**Hypertension**	66%	65%	81%	0.35	0.11
**Diabetes**	23%	15%	14%	0.11	0.34
**Albumin-creatinine ratio (median, IQR)**	10 (7-18)	8 (6-12)	9 (7-20)	0.23	0.86
**Baseline Kidney Function**					
**Creatinine Clearance (mean)**	92 ± 36	93 ± 34	103 ± 40	0.28	0.12
**eGFR (cysC) (mean)**	69 ± 22	69 ± 21	79 ± 25	0.20	0.08
**eGFR (Scr) (mean)**	73 ± 20	74 ± 19	78 ± 22	0.51	0.38
**Non-Whites**					
**N**	310	39	3	-	-
**Age**	65 ± 11	64 ± 9	57 ± 7	0.27	0.13
**Male**	77%	82%	33%	0.14	0.13
**Body Mass Index**	28 ± 6	29 ± 5	38 ± 8	0.02	0.02
**Hypertension**	78%	77%	100%	0.65	0.47
**Diabetes**	36%	31%	33%	0.82	0.71
**Albumin-creatinine ratio (median, IQR)**	11 (7-36)	11 (6-16)	49 (20-78)	0.35	0.15
**Baseline Kidney Function**					
**Creatinine Clearance (mean)**	90 ± 37	92 ± 40	105 ± 26	0.66	0.39
**eGFR (cysC) (mean)**	72 ± 25	75 ± 22	72 ± 21	0.83	0.84
**eGFR (Scr) (mean)**	76 ± 27	82 ± 24	71 ± 10	0.29	0.59

P values are obtained using Kruskal-Wallis rank or the Mann-Whitney test for continuous variables, Fisher's exact test or Chi-square test for categorical variables.

*UMOD *genotype was associated with urine uromodulin concentration - the median was more than 50% higher in the G/G group compared with the T/T genotype, and heterozygotes (G/T) had intermediate levels (Table 2). In both unadjusted and adjusted linear regressions, the T/T genotype had significantly lower uromodulin levels than the common G/G genotype, but differences between the G/T and G/G genotypes were not statistically significant. These findings were essentially unchanged when we restricted to White participants.

**Table 2:** Association of UMOD Genotype at rs12917707 and *UMOD *Levels

Characteristics	G/G (n = 48)	G/T (n = 48)	T/T (n = 24)	P value
**Overall**				

**UMOD (median IQR)**	142 (87-249)	128 (64-207)	83 (33-159)	0.11
**Linear Regression (log transformed) (β Coefficient, p-value)**				
**Unadjusted**	Reference	-0.2	-0.7	-
		p = 0.36	p = 0.002	
Adjusted *	Reference	-0.2	-0.8	-
		p = 0.38	p = 0.001	
**Restricted to Whites**				
**UMOD (median IQR)**	152 (105-268)	129 (67-207)	97 (33-170)	0.23
**Linear Regression (log transformed) (β Coefficient, p-value)**				
**Unadjusted**	Reference	-0.4	-0.8	-
		p = 0.09	p = 0.001	
Adjusted *	Reference	-0.4	-0.9	-
		p = 0.10	p < 0.001	

P values are obtained using Kruskal-Wallis rank test for mean comparison and non-parametric median test for median comparison.

*Adjusted for age, sex, race, BMI, diabetes, and hypertension

As a result of the matched design, the 102 cases and 94 controls with uromodulin measures, matches were similar in age, sex, and race (Table 3). Although all cases and controls had baseline CrCl > 70 ml/min, CrCl was significantly lower at baseline among future cases than controls. However, the average change in CrCl was more than 2-fold among cases compared with controls. Cases also had a more than 3-fold increase in serum creatinine and a near doubling in eGFR decline compared with controls. Uromodulin levels were similar between cases and controls, regardless of whether the comparison was made of uromodulin concentrations, UCR, or by 24-hour uromodulin (Table 3). Using conditional logistic regression, each higher SD of baseline uromodulin or UCR had no significant association with odds of incident CKD. We also compared the distribution of quartiles of uromodulin concentrations and UCR by case-control status; no differences were observed (all p-values > 0.20). Finally, we compared the UMOD genotypes among cases and controls. There was no significant difference in the distribution (p-value = 0.33), but the protective T/T genotype appeared less frequent in cases than controls (2% vs. 5%).

**Table 3:** Association of Baseline Uromodulin with Incident CKD in a Nested Case-Control Design

	Cases† (n = 102)	Controls (n = 94)	P value
**Age**	66 ± 10	66 ± 9	0.60
**Male**	85%	86%	0.86
**Race**			0.90
**Whites**	57%	54%	
**Blacks**	20%	20%	
**Other**	23%	26%	
**Body Mass Index (BMI) (mean)**	30 (6.4)	29 (5.3)	0.25
**Diabetes**	34%	27%	0.24
**SBP**	134 (19.2)	134 (21.3)	0.99
**DBP**	76 (10.6)	77 (11.5)	0.46
**Smoking status (Y)**	20%	13%	0.21
**ACE/ARB use**	63%	50%	0.07
**Satins use**	68%	79%	0.08
**Albumin-creatinine ratio (median)**	9 (7-25)	8 (5-13)	0.53
**Baseline CrCl (mean)**	93 ± 23	113 ± 30	<0.001
**Final CrCl (mean)**	52 ± 13	95 ± 25	<0.001
**Change in CrCl (mean)**	-42 ± 20	-18 ± 25	<0.001
**Baseline serum creatinine**	1.08	0.96	<0.001
**Final serum creatinine**	1.44	1.06	<0.001
**Change in creatinine**	0.36	0.10	<0.001
**Baseline eGFRcr**	74	85	<0.001
**Final eGFRcr**	55	75	<0.001
**Change in eGFRcr**	19	10	<0.001
Uromodulin concentration (mg/dL)* (median, IQR)	44 (26-85)	48 (23-80)	0.88
**Urine uromodulin-creatinine ratio (mg/g) (median, IQR)**	51 (26-75)	47 (23-83)	0.34
**24-hour urine uromodulin (mg) (median, IQR)**	71 (35-110)	77 (39-121)	0.75
**Odds of Incident CKD**			
**Uromodulin concentration (per SD)**	1.05 (0.73-1.51)	Reference	0.78
**Urine uromodulin-creatinine ratio (per SD)**	1.05 (0.77-1.43)	Reference	0.76
**24-hour urine uromodulin concentration (per SD)**	1.01 (0.74-1.36)	Reference	0.97
**Genotype Frequencies**:			0.33
**G/G genotype frequency**	79%	72%	
**G/T genotype frequency**	19%	22%	
**T/T genotype frequency**	2%	5%	

†cases and controls matched on age, sex, and race

*UMOD non-detectables set as zero; no difference when excluded non-detectables.

CrCl = creatinine clearance

IQR = interquartile range; SD = standard deviation

## Discussion

The role of uromodulin in the onset and progression of kidney disease is currently of great interest in clinical nephrology. Disorders in the *UMOD *gene lead to the early onset of severe kidney disease.[3] A more common variant in the *UMOD *genotype has been associated with lower risk of CKD in persons of European descent.[1] Higher urine concentrations of uromodulin were associated with subsequent development of CKD in the FHS, a community-based, predominantly White study.[5] However, in this study from the Heart and Soul cohort of persons with established CAD, we found no association between urine concentrations of uromodulin or total daily uromodulin and incident CKD. Yet, *UMOD *genotype was associated with urine uromodulin levels in a similar direction to the FHS and ARIC studies.

Several potential reasons could explain why findings in this study regarding the association of uromodulin and CKD differ from our prior work in FHS. Most importantly, the progression of CKD may be proportionally related to different mechanisms between persons with and without CAD. Among the Heart and Soul cohort, kidney function decline may be caused predominantly by atherosclerotic mechanisms. Whatever role urine uromodulin concentrations may have in promoting kidney decline may be less influential relative to the role of microvascular disease. A second possibility would be measurement error, as we developed our own uromodulin assay. However, our replication of the association between *UMOD *genotype and urine levels suggests that our assay functioned accurately. In addition, chance could have led us to miss an association between urine uromodulin levels and incident CKD, but our findings were robustly null and the confidence interval around the odds ratio for incident CKD was relatively narrow (0.71-1.26), and clearly excluded the 71% increased odds observed in FHS. Furthermore, our cohort was a mix of Whites and non-Whites and other factors may predominate in non-Whites [14]. Finally, cases and controls differed in their baseline CrCl, while a similar imbalance of baseline eGFR was present in the FHS analysis.

Despite the null result of our primary hypothesis, this study has several strengths. To our knowledge, this is only the second study to evaluate uromodulin concentrations as a predictor of incident CKD; although an exciting potential biomarker, urine uromodulin levels should be evaluated in multiple diverse settings to determine their potential value in clinical medicine. The Heart and Soul cohort had several unique attributes, including measured kidney function by CrCl on two occasions, the availability of stored urine and genetic material, and a 5-year follow-up interval. The primary limitation was a limited sample size to evaluate homozygotes for the minor allele; however, this group has a population prevalence of only 3-4%, so much larger studies are required to characterize them further. A second limitation is the sample size of our nested case-control design; yet, the confidence intervals do not suggest that we would have missed an important finding by chance alone. A third limitation could be errors in the urine collections leading to biased estimates of CrCl; however, cases had much larger changes in creatinine than controls, as well.

## Conclusions

In summary, in this cohort of persons with established coronary artery disease, we replicated the association of UMOD genotype with urine levels but found no association between urine uromodulin concentrations and incident CKD. The differences from the initial report suggest that differences in population, urine collection and measurement may influence the results. Future study will be required to determine the value of urine uromodulin as a prognostic biomarker for discriminating risk for the onset and progression of CKD.

## Competing interests

The authors declare that they have no competing interests.

## Authors' contributions

MGS: made substantial contributions to conception and design, acquisition of data, analysis and interpretation of data; involved in drafting the manuscript and revising it critically for important intellectual content; and has given final approval of the version to be published.**YL**: analysis and interpretation of data; involved in drafting the manuscript and revising it critically for important intellectual content; and has given final approval of the version to be published.**CF**: made substantial contributions to conception and design; involved in drafting the manuscript and revising it critically for important intellectual content; and has given final approval of the version to be published.**JC**: made substantial contributions to conception and design; involved in drafting the manuscript and revising it critically for important intellectual content; and has given final approval of the version to be published.**CG**: made substantial contributions to conception and design, acquisition of data; involved in drafting the manuscript and revising it critically for important intellectual content; and has given final approval of the version to be published.**MW**: made substantial contributions to conception and design, acquisition of data, analysis and interpretation of data; involved in drafting the manuscript and revising it critically for important intellectual content; and has given final approval of the version to be published.

## Pre-publication history

The pre-publication history for this paper can be accessed here:

http://www.biomedcentral.com/1471-2369/12/2/prepub

## References

[B1] Kottgen A Glazer NL Dehghan A Hwang SJ Katz R Li M Yang Q Gudnason V Launer LJ Harris TB Multiple loci associated with indices of renal function and chronic kidney disease Nat Genet 2009 42 5 376 384 10.1038/ng.568 PMC3039280 19430482

[B2] Kottgen A Pattaro C Boger CA Fuchsberger C Olden M Glazer NL Parsa A Gao X Yang Q Smith AV New loci associated with kidney function and chronic kidney disease Nat Genet 42 5 376 384 10.1038/ng.568 20383146 PMC2997674

[B3] Bleyer AJ Hart TC Shihabi Z Robins V Hoyer JR Mutations in the uromodulin gene decrease urinary excretion of Tamm-Horsfall protein Kidney Int 2004 66 3 974 977 10.1111/j.1523-1755.2004.00845.x 15327389

[B4] Sedor JR Uromodulin and translational medicine: will the SNPs bring zip to clinical practice? J Am Soc Nephrol 21 2 204 206 10.1681/ASN.2009121283 20110381

[B5] Kottgen A Hwang SJ Larson MG Van Eyk JE Fu Q Benjamin EJ Dehghan A Glazer NL Kao WH Harris TB Uromodulin levels associate with a common UMOD variant and risk for incident CKD J Am Soc Nephrol 2010 21 2 337 344 10.1681/ASN.2009070725 19959715 PMC2834540

[B6] Shlipak MG Katz R Kestenbaum B Fried LF Siscovick D Sarnak MJ Clinical and subclinical cardiovascular disease and kidney function decline in the elderly Atherosclerosis 2008 204 1 298 303 10.1016/j.atherosclerosis.2008.08.016 18848325 PMC2696894

[B7] Elsayed EF Tighiouart H Griffith J Kurth T Levey AS Salem D Sarnak MJ Weiner DE Cardiovascular disease and subsequent kidney disease Arch Intern Med 2007 167 11 1130 1136 10.1001/archinte.167.11.1130 17563020

[B8] Peralta CA Katz R Madero M Sarnak M Kramer H Criqui MH Shlipak MG The differential association of kidney dysfunction with small and large arterial elasticity: the multiethnic study of atherosclerosis Am J Epidemiol 2009 169 6 740 748 10.1093/aje/kwn392 19131564 PMC2727212

[B9] Madero M Peralta CA Wassel Fyr CL Najjar SS Sutton-Tyrrell K Fried L Canada RB Newman A Shlipak MG Sarnak M Cystatin C associates with arterial stiffness in older adults J Am Soc Nephrol 2009 20 5 1086 1093 10.1681/ASN.2008030318 19357259 PMC2678034

[B10] Whooley MA de Jonge P Vittinghoff E Otte C Moos R Carney RM Ali S Dowray S Na B Feldman MD Depressive symptoms, health behaviors, and risk of cardiovascular events in patients with coronary heart disease JAMA 2008 300 20 2379 2388 10.1001/jama.2008.711 19033588 PMC2677371

[B11] Levey AS Bosch JP Lewis JB Greene T Rogers N Roth D A more accurate method to estimate glomerular filtration rate from serum creatinine: a new prediction equation. Modification of Diet in Renal Disease Study Group Annals of Internal Medicine 1999 130 6 461 470 10075613 10.7326/0003-4819-130-6-199903160-00002

[B12] Stevens LA Coresh J Schmid CH Estimating GFR using serum cystatin C alone and in combination with serum creatinine: a pooled analysis of 3,418 individuals with CKD Am J Kidney Dis 2008 51 395 406 10.1053/j.ajkd.2007.11.018 18295055 PMC2390827

[B13] Lau WH Leong WS Ismail Z Gam LH Qualification and application of an ELISA for the determination of Tamm Horsfall protein (THP) in human urine and its use for screening of kidney stone disease Int J Biol Sci 2008 4 4 215 222 18695745 10.7150/ijbs.4.215 PMC2500153

[B14] Kao WH Klag MJ Meoni LA Reich D Berthier-Schaad Y Li M Coresh J Patterson N Tandon A Powe NR MYH9 is associated with nondiabetic end-stage renal disease in African Americans Nat Genet 2008 40 10 1185 1192 10.1038/ng.232 18794854 PMC2614692

